# A commercial line probe assay for the rapid detection of rifampicin resistance in *Mycobacterium tuberculosis*: a systematic review and meta-analysis

**DOI:** 10.1186/1471-2334-5-62

**Published:** 2005-07-28

**Authors:** Maureen Morgan, Shriprakash Kalantri, Laura Flores, Madhukar Pai

**Affiliations:** 1 Division of Epidemiology, School of Public Health, University of California, Berkeley, U.S.A; 2 Department of Medicine, Mahatma Gandhi Institute of Medical Sciences, Sevagram, India; 3 Departamento de Biomedicina Molecular, CINVESTAV-IPN, Mexico; 4 Division of Pulmonary & Critical Care Medicine, San Francisco General Hospital, University of California, San Francisco, U.S.A

## Abstract

**Background:**

*Mycobacterium tuberculosis *is a leading cause of death worldwide. In multi-drug resistant tuberculosis (MDR-TB) infectiousness is frequently prolonged, jeopardizing efforts to control TB. The conventional tuberculosis drug susceptibility tests are sensitive and specific, but they are not rapid. The INNO-LiPA Rif. TB ^® ^(LiPA) is a commercial line probe assay designed to rapidly detect rifampicin resistance, a marker of MDR-TB. Although LiPA has shown promising results, its overall accuracy has not been systematically evaluated.

**Methods:**

We did a systematic review and meta-analysis to evaluate the accuracy of LiPA for the detection of rifampicin-resistant tuberculosis among culture isolates and clinical specimens. We searched Medline, Embase, Web of Science, BIOSIS, and Google Scholar, and contacted authors, experts and the manufacturer. Fifteen studies met our inclusion criteria. Of these, 11 studies used culture isolates, one used clinical specimens, and three used both. We used a summary receiver operating characteristic (SROC) curve and Q* index to perform meta-analysis and summarize diagnostic accuracy.

**Results:**

Twelve of 14 studies that applied LiPA to isolates had sensitivity greater than 95%, and 12 of 14 had specificity of 100%. The four studies that applied LiPA directly to clinical specimens had 100% specificity, and sensitivity that ranged between 80% and 100%. The SROC curve had an area of 0.99 and Q* of 0.97.

**Conclusion:**

LiPA is a highly sensitive and specific test for the detection of rifampicin resistance in culture isolates. The test appears to have relatively lower sensitivity when used directly on clinical specimens. More evidence is needed before LiPA can be used to detect MDR-TB among populations at risk in clinical practice.

## Background

Tuberculosis (TB) continues to be a major public health problem, particularly in developing countries. The WHO estimates that one third of the world's population is infected with *Mycobacterium tuberculosis*, the causative agent of TB. There were an estimated 8.3 million new active cases and 1.8 million deaths from TB in the year 2000, making it the second greatest killer among infectious diseases worldwide [1].

The prevalence of multidrug-resistant TB (MDR-TB), defined as resistance to at least rifampicin (RIF) and isoniazid (INH), is rising in a number of geographic regions. According to a recent WHO report [1], the median prevalence of MDR-TB is 1% (range 0%–14.1%) among new cases and 9.3% (range 0%–48%) among previously treated cases. Rapid identification is essential for effective treatment and control of MDR-TB. Conventional methods of drug susceptibility testing (DST) include solid media-based methods such as the proportion, absolute concentration, and resistance ratio methods. These can take up to 12 weeks to produce definitive results, leading to prolonged infectiousness [2]. Liquid media-based tests, such as the BACTEC^®^, MB/BacT^®^, ESP^® ^and MGIT^® ^systems, are more rapid, but also more costly and require sophisticated laboratories and trained personnel [2].

Rifampicin works by binding to the beta-subunit of the RNA polymerase (coded for by the *rpo*B gene), inhibiting protein transcription [3]. DNA sequencing studies have shown that greater than 95% of the RIF-resistant strains have mutations within an 81 base pair hot-spot region (codons 507–533) of the *rpo*B gene [4]. Though more than 50 mutations within this region have been characterized by automated DNA sequencing, the majority involve point mutations at codons 516, 526, or 531 [5]. It is estimated that more than 90% of RIF-resistant TB is also resistant to INH, making RIF-resistance a good surrogate marker for MDR-TB [4,6]. The above observations have lead to the recent development of several genotypic methods for rapidly detecting RIF-resistance conferring mutations, including DNA sequencing, line probe assay, single-strand conformation polymorphism, DNA microarrays, RNA/RNA mismatch, and molecular beacons [2].

The commercially available INNO-LiPA Rif. TB kit (Innogenetics, Zwijndrecht, Belgium) is a line probe assay (LiPA) able to identify the *M. tuberculosis *complex and simultaneously detect genetic mutations in the *rpo*B gene region related to RIF-resistance [7]. The LiPA kit contains 10 oligonucleotide probes (one specific for the *M. tuberculosis *complex, five overlapping wild-type S probes, and four R probes for detecting specific mutations of resistant genotypes) immobilized on nitrocellulose paper strips [7].

LiPA is performed by extracting DNA from cultures or directly from clinical samples and amplifying the RIF- resistance-determining region of the *rpo*B gene using PCR. Biotinylated PCR products are then hybridized with the immobilized probes, and results are determined by colorimetric development. The *M. tuberculosis *isolate is considered RIF susceptible if all of the wild-type S probes give a positive signal and all of the R probes react negatively. RIF resistance is indicated by absence of one or more wild-type S probes. When RIF resistance is due to one of the four most frequently observed mutations, a positive reaction is obtained with one of the four R probes [7].

A number of studies [3,5,7-19] have evaluated the diagnostic accuracy of LiPA for detecting RIF resistance in diverse geographic settings. We conducted a systematic review and meta-analysis to evaluate the overall accuracy of line probe assay in the detection of RIF-resistant TB.

## Methods

### Search strategy

We searched the following databases for retrieving articles and abstracts based on primary studies: Pubmed, Embase, Biosis, Web of Science (all 1990–2004), and Google Scholar (December 2004) using the keywords and search terms "Tuberculosis", "Mycobacterium tuberculosis", "Tuberculosis, Multidrug-Resistant", "Drug Resistance", "Drug Resistance, Bacterial", "rifampicin", "Rifampin", "mutation", "mutant", "rpob", "rpob gene", "line probe", "line probe assay", "LiPA", and "INNO-LiPA". We also contacted authors and experts, including the manufacturer of the commercial INNO-LiPA Rif.Tb kit, for lists of references and unpublished data, and reviewed citations of relevant primary studies and review articles.

#### Study selection

We identified results from all primary studies evaluating the accuracy (sensitivity and specificity) of line probe assay (specifically, the commercial INNO-LiPA Rif. TB kit) for rapid detection of RIF-resistant TB in clinical specimens or isolates. Titles and/or abstracts of all citations were screened independently by two reviewers (MM and SK), with 85% agreement on articles warranting full text review. Differences between reviewers were reconciled by consensus, and the full text of all relevant studies was evaluated.

We included studies that met the following pre-determined criteria: (i) comparison of INNO-LiPA with a reference standard (including proportion method, radiometric BACTEC 460 method, and minimum inhibitory concentration method), (ii) evaluation of a minimum of ten RIF-sensitive and ten RIF-resistant samples.

Although our initial search had no language restrictions, studies not available in either English or Spanish language were excluded from the data extraction process.

#### Data extraction and assessment of study quality

All included articles were assessed by one reviewer (MM), who extracted data using a piloted data extraction form. A second reviewer (LF) independently extracted data from a subset (five out of fifteen) of the included studies, with an inter-rater agreement between the two reviewers of 80% for sensitivity and specificity data. Discrepancies between reviewers were reconciled by consensus. Extracted data included the reference standard used, type of sample (clinical specimen *vs*. isolate), outcome data (sensitivity and specificity as determined by comparison with the reference standard), and proportion of RIF-resistant samples that were determined to be MDR-TB.

We assessed study quality using the following criteria, based on the QUADAS criteria [20] for assessment of quality of diagnostic studies: (i) prospective enrolment of consecutive patients, (ii) comparison with an appropriate reference standard, (iii.) blind and independent comparison of the index test (LiPA) with a reference standard, and (iv) verification (partial or complete) of LiPA results by reference standards.

#### Data synthesis and meta-analysis

We used standard methods for diagnostic meta-analysis [23,24], and performed data analysis using the Meta-Disc (version 1.1.1) software [23].

We focused on sensitivity and specificity as measures of diagnostic accuracy of LiPA. These were computed by creating a two by two table of LiPA RIF-susceptibility results against reference standard RIF-susceptibility results for each study and cross-tabulating. Sensitivity (true positive rate [TPR]) in this case is defined as the proportion of samples determined to be RIF-resistant by a reference standard correctly identified as RIF-resistant by LiPA. Specificity (true negative rate or 1-false positive rate [FPR]) is defined as the proportion of samples determined to be RIF-sensitive by a reference standard correctly identified as RIF-sensitive by LiPA. We created forest plots to display estimates of accuracy and examine the heterogeneity (between-study variability) of the summary measures of sensitivity and specificity.

We summarized the joint distribution of TPR and FPR with a summary receiver operating characteristic (SROC) curve. SROC curves used in analyses of diagnostic accuracy are intended to represent the relationship between TPR and FPR across studies when test performance is evaluated at varying diagnostic thresholds [24]. Each study is a separate unit of analysis and contributes an estimate of TPR and FPR. Overall diagnostic performance of a test can be judged by the position and appearance of the SROC curve, which is fitted by using a regression model proposed by Moses *et al *[25]. The area under the curve (AUC) represents an overall summary measure of the curve and the test's overall ability to accurately distinguish cases from non-cases. The Q* index, the highest point on the SROC curve that intersects the anti-diagonal, represents a summarization of test performance where sensitivity and specificity are equal (so the probability of an incorrect test result is the same for cases and non-cases). An AUC of one represents perfect discriminatory ability, while a Q* index of one represents perfect accuracy [24].

## Results

### Description of included studies

**Figure 1:**
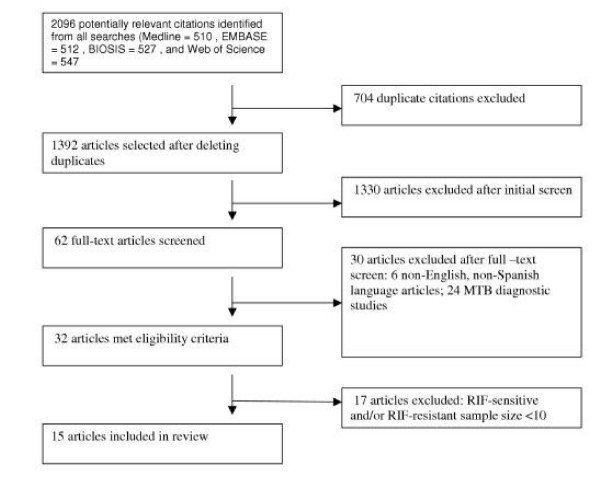
Study selection process and reasons for exclusion of studies.

Figure 1 illustrates the study selection process. Fifteen articles [3,5,7-19], all reporting results of primary studies, met eligibility criteria and are included in this review.

Table 1 describes the characteristics and outcomes of the 15 included studies. Three studies [10,11,19] are listed twice in order to describe the outcome of a subgroup analysis of LiPA applied directly to clinical specimens. All studies were published between 1995 and 2004 and used the commercial INNO-LiPA Rif. TB kit according to the manufacturer's instructions. Eleven studies [3,5,7,12-18] tested LiPA exclusively on culture isolates, one study [8] tested LiPA directly on clinical specimens, and three studies [10,11,19] tested LiPA on both isolates and clinical specimens. Clinical specimens included sputum, bronchial aspirate, urine, tissue biopsy, cerebrospinal fluid, feces, skin exudates, and gastric juice aspirate.

**Table 1:** Description of studies included in meta-analysis.

**Author (year)**	**Country**	**Reference Test**	**Blinded to reference test?**	**Sample**	**Sample size (# resistant / # sensitive)**	**Sensitivity (95% CI)**	**Specificity (95% CI)**
Ahmad (2002)	Kuwait	BACTEC 460	Not Specified	Isolate	29/12	0.97 (.82–1.0)	1.0 (.74–1.0)
De Oliveira (1998)	Brazil	Proportion	Not Specified	Isolate	113/15	0.97 (.92–.99)	1.0 (.78–1.0)
Gamboa (1998)	Spain	BACTEC 460	Not Specified	Isolate	46/13	1.0 (.92–1.0)	1.0 (.75–1.0)
Hirano (1999)	Japan	Proportion	Not Specified	Isolate	90/26	0.92 (.85–.97)	1.0 (.87–1.0)
Johansen (2003)	Denmark	BACTEC 460	Not Specified	Isolate	35/24	0.97 (.85–1.0)	1.0 (.86–1.0)
Jureen (2004)	Sweden	BACTEC 460	Not Specified	Isolate	27/26	1.0 (.87–1.0)	0.92 (.75–.99)
Lemus (2004)	Belgium	BACTEC 460, Proportion	Yes	Isolate	10/10	1.0 (.69–1.0)	1.0 (.69–1.0)
Rossau (1997)	Belgium	Proportion	Not Specified	Isolate	203/61	0.98 (.95–.1.0)	1.0 (.94–1.0)
Sintchenko (1999)	Australia	BACTEC 460	Not Specified	Isolate	22/11	0.96 (.77–1.0)	1.0 (.72–1.0)
Somoskovi (2003)	USA	Proportion	Not Specified	Isolate	64/37	0.95 (.87–.99)	1.0 (.91–1.0)
Srivastava (2004)	India	MIC	Not Specified	Isolate	45/10	0.82 (.68–.92)	1.0 (.69–1.0)
Tracevska (2002)	Latvia	BACTEC 460	Not Specified	Isolate	34/19	1.0 (.90–1.0)	1.0 (.82–1.0)
Traore (2000)	Belgium	Proportion	Not Specified	Isolate	266/145	0.99 (.96–1.0)	1.0 (.98–1.0)
Watterson (1998)	England	BACTEC 460, Proportion	Not Specified	Isolate	16/16	1.0 (.80–1.0)	0.94 (.70–1.0)
De Beenhouwer (1995)	Belgium	Proportion	Not Specified	Clinical Specimen	21/46	0.91 (.70–1.0)	1.0 (.92–1.0)
Gamboa (1998)	Spain	BACTEC 460	Not Specified	Clinical Specimen	46/13	0.98 (.89–1.0)	1.0 (.75–1.0)
Johansen (2003)	Denmark	BACTEC 460	Not Specified	Clinical Specimen	26/21	1.0 (.87–1.0)	1.0 (.84–1.0)
Watterson (1998)	England	BACTEC 460, proportion	Yes	Clinical Specimen	10/24	0.80 (.44–.98)	1.0 (.86–1.0)

The 15 studies evaluated 1738 specimens (mean 91; range 20 to 411), 1164 (67%) of which were RIF-resistant. Twelve of the 15 studies include a greater number of RIF-resistant than RIF-sensitive strains (mean 87 and 36 respectively). Six studies [3,7-9,15,18] used proportion method, six studies [5,10-12,14,17] used BACTEC 460, two studies [13,19] used both proportion method and BACTEC 460, and one study [16] used minimum inhibitory concentration (MIC) method as the reference test. Only two studies [13,19] explicitly reported blinding researchers to the results of the reference standard and/or LiPA. None of the studies prospectively enrolled consecutive patients, and all had complete verification of LiPA with a reference standard.

### Accuracy of LiPA in isolates

**Figure 2:**
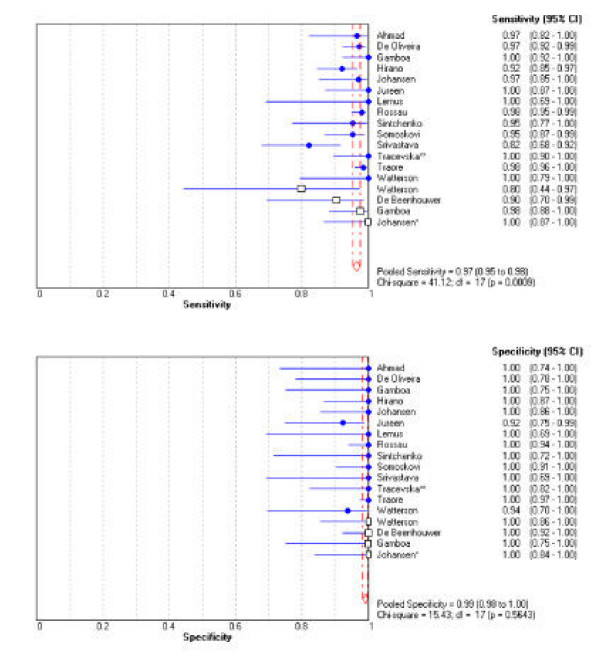
**Forrest plots of sensitivity and specificity. **The point estimates of sensitivity and specificity from each study are shown as solid circles (culture isolates) and open rectangles (clinical specimens). Error bars are 95% confidence intervals (CI).

**Figure 3:**
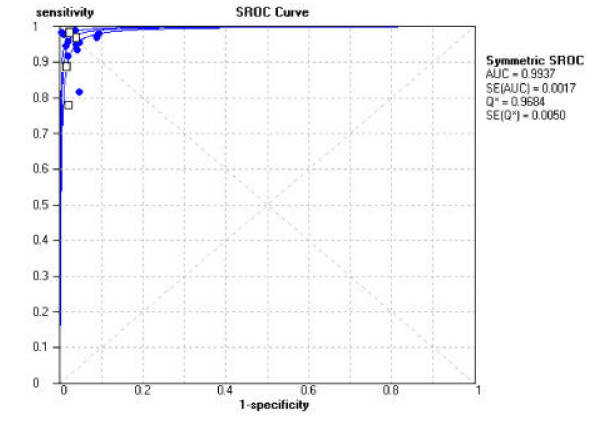
**Summary Receiver Operator Curve (SROC) plot for line probe assay. **Each solid circle (culture isolate) and open rectangle (clinical specimen) represents each study in the meta-analysis. The curve is the regression line that summarizes the overall diagnostic accuracy. SROC: summary receiver operating characteristic; AUC: area under the curve; SE(AUC): standard error of AUC; Q*: an index defined by the point on the SROC curve where the sensitivity and specificity are equal, which is the point closest to the top-left corner of the ROC space; SE(Q*): standard error of Q* index.

Figure 2 illustrates a forest plot of estimates of sensitivity and specificity based on results of the 15 included studies and stratified by type of sample (isolate *vs*. clinical specimen). Figure 3 is a SROC curve of the same data. As seen in figure 2, of the 14 studies that applied LiPA to isolates, sensitivity ranged from 82% to 100%, and specificity ranged from 92% to 100%. Twelve studies [5,7,9-15,17-19] reported sensitivity >= 95%, and five of these studies [10,12,13,17,19] reported sensitivity of 100%. With the exception of two [12,19], all studies reported specificity of 100%.

The SROC curve, figure 3, shows an area of 0.99 and Q* of 0.97, indicating a high level of overall accuracy.

### Subgroup analysis of accuracy of LiPA in clinical specimens

As illustrated in figure 2, of the four studies that tested LiPA directly on clinical specimens [8,10,11,19], sensitivity estimates, although more variable than specificity, are consistently high (80% to 100%) with one study [11] reporting a sensitivity of 100%. The specificity estimates for all four studies are 100%.

Although still consistently high, sensitivity appears to be lower overall in clinical specimens than isolates. Additionally, one study [11] explicitly stated that 13 of the 60 samples tested were indeterminate due to failure at the PCR stage, making it impossible to perform LiPA. These are excluded from measures of sensitivity and specificity, indicating that the overall accuracy of LiPA applied to clinical specimens may be inflated in this study (and possibly others if they experienced similar indeterminate results that went unreported) when compared with performance in an actual clinical setting.

### Rifampicin-resistance as a marker of MDR-TB

Four studies [5,8,12,18] determined the number of RIF-resistant samples that were also INH-resistant, thereby meeting the criteria for MDR-TB. On average, 91% of RIF-resistant samples were also INH-resistant.

## Discussion

### Principle findings

This meta-analysis suggests that the LiPA assay is highly sensitive and specific for detecting rifampicin-resistant TB both in culture isolates and, to a slightly lesser degree, clinical specimens. The majority of studies had sensitivity of 95% or greater, and nearly all were 100% specific.

Despite variations in patient populations, all 15 studies yielded consistently high estimates of sensitivity and specificity, so heterogeneity was not a concern in this meta-analysis [26].

### Clinical implications

The currently employed DST methods typically delay the diagnosis of MDR-TB by at least one to two months. A more rapid method is needed to allow timely diagnosis and initiation of effective treatment. This meta-analysis demonstrates that LiPA yielded high overall sensitivity and specificity with a maximum joint sensitivity and specificity of 97% (based on the Q* index). The test may thus have a potential role in ruling in and ruling out the diagnosis of RIF-resistance. For example, assuming that 5% of TB patients in a clinical setting have RIF-resistant TB, a positive LiPA result (inferring RIF-resistance) would yield a positive predictive value of 83%, while a negative LiPA result would yield a negative predictive value of 99%. These test results would lead to a clinically meaningful increase in the probability of RIF-resistance from 5% to 83% if a test is positive, while a negative test would virtually rule out RIF-resistance. Because the test has a high sensitivity, a negative result would effectively rule out the probability of drug resistance. Similarly, because the test has a high specificity, a positive result would rule in drug resistance. However, the diagnostic accuracy of LiPA needs to be interpreted cautiously in low prevalence areas. For example, if the baseline prevalence of rifampicin resistance is 1%, a positive test would translate into a positive predictive value of only 66%, i.e. one false positive test for every two true positives. As with any diagnostic test, if used judiciously (ie in patients suspected of having MDR-TB, thereby raising the pretest probability) the accuracy of LiPA could be maintained even in low prevalence regions.

Because patients with MDR-TB are more likely to be put on an effective drug therapy regimen if the drug resistance is quickly detected, and thus are less likely to transmit MDR-TB to the community, the benefits of early detection of drug resistance can be substantial. A positive test in a high prevalence setting can lead to a highly meaningful shift from pre-test to post-test probability and thus may facilitate better outcomes.

LiPA has shown a high degree of accuracy when used on culture isolates, but this requires 2–6 weeks for primary isolation. Only four studies applied LiPA directly to clinical specimens, resulting in slightly more variation in the degree of accuracy than those studies using isolates. Additional research is needed to establish the accuracy of LiPA applied to clinical specimens, but the preliminary studies suggest that LiPA may help diagnose RIF-resistant TB within 24–48 hours of sample collection.

The cost of the commercial LiPA kit is $45 per sample tested. When additional costs for import and transport are taken into account, the actual cost per sample is as high as $116 [27]. Though this may be prohibitively expensive for routine use in the regions of the world with the highest prevalence and incidence of TB and MDR-TB, judicious use of LiPA for patients with a high likelihood of MDR-TB (for example, smear-positive patients with treatment failure or relapse from high incidence areas and/or previously treated patients) may be possible, particularly when weighed against the costs of undetected drug resistant TB. An additional challenge to widespread implementation of LiPA is the requirement of a lab with technical expertise in performing PCR.

### Strengths and weaknesses of the review

This review has several strengths. We performed a comprehensive search for literature by exploring five electronic databases and by contacting authors, experts, and the manufacturer of the reviewed index test. Study selection was conducted independently by two reviewers, as was data extraction and quality review for a subset of included studies, and disagreements were resolved with discussion. We performed meta-analyses in accordance with published guidelines [21,22].

This review has some limitations. We excluded studies not available in English or Spanish language, which could introduce publication bias. However, a review of the abstracts of these papers suggests that the overall results are similar to the results in the included English and Spanish language studies. Publication bias may also be introduced by inflation of diagnostic accuracy estimates since studies that report positive results are more likely to be accepted for publication. The studies included in this meta-analysis apply LiPA to a total of 1738 MTB positive samples, of which 1164 are RIF-resistant. This prevalence of 67% differs significantly from the prevalence of MDR-TB seen in routine clinical practice settings, even in high prevalence regions such as Estonia (14.1%), Henan Province in China (10.8%), Latvia (9%), and the Russian oblasts of Ivanovo (9%) and Tomsk(6.5%) [1]. Because the specimens analyzed in the studies are not a true representation of specimens that a TB laboratory would actually receive, estimates of sensitivity and specificity may be inflated. Finally, estimates of sensitivity and specificity may be inflated in these studies due to exclusion of indeterminate results from measures of accuracy if failure occurred at the PCR stage, which precludes performance of LiPA on the specimen or isolate.

### Implications for research

Additional studies are needed to establish the accuracy of LiPA used directly on clinical specimens. Study design should include selection of sputum samples from patients suspected of having MDR-TB (ie patients with treatment failure or relapse from high incidence areas and/or previously treated patients). Indeterminate results, the proportion of RIF-resistant specimens that meet MDR-TB criteria, patients' sputum smear status, and turnaround time for diagnosis should be reported.

Studies are also needed to establish clinical usefulness of rapid diagnosis of RIF-resistant TB in terms of the effect on clinical outcomes and TB transmission rates. Finally, studies are needed to establish the cost benefit advantages of LiPA over conventional DST.

## Conclusion

Line probe assay has been shown to be highly sensitive and specific in the detection of rifampicin-resistant TB when used on culture isolates. There is a paucity of data on application of this test directly to clinical specimens, although based on a small number of studies, the test appears to be less promising. The cost of the kit may render the test impractical for widespread use in those regions of the world most affected by MDR-TB and most in need of a method for its rapid diagnosis. However if further studies indicate that line probe assay consistently and accurately detects RIF-resistant TB when applied directly to clinical specimens, it could be a useful test in select patient populations in which MDR-TB is strongly suspected.

## Competing interests

The author(s) declare that they have no competing interests.

## Authors' contributions

MM designed the study, searched the databases, extracted the data, analyzed the results and wrote the manuscript. SK helped with study design, searching the databases, writing and revising the manuscript, and served as a second reviewer in screening articles for inclusion. MP formulated the research question, helped with study design, database searches, analysis, and in revising the manuscript. LF helped design the data abstraction form, provided critical input in laboratory associated issues and served as a second reviewer in extracting data. All authors read and approved the final manuscript.

## Pre-publication history

The pre-publication history for this paper can be accessed here:


